# Cell apoptosis, autophagy and necroptosis in osteosarcoma treatment

**DOI:** 10.18632/oncotarget.8206

**Published:** 2016-03-19

**Authors:** Jing Li, Zuozhang Yang, Yi Li, Junfeng Xia, Dongqi Li, Huiling Li, Mingyan Ren, Yedan Liao, Shunling Yu, Yanjin Chen, Yihao Yang, Ya Zhang

**Affiliations:** ^1^ Bone and Soft Tissue Tumors Research Center of Yunnan Province, Department of Orthopaedics, the Third Affiliated Hospital of Kunming Medical University (Tumor Hospital of Yunnan Province), Kunming, Yunnan, China; ^2^ State Key Laboratory of Protein and Plant Gene Research, College of Life Sciences, Peking University, Beijing, China; ^3^ Department of Oncology, Kunming General Hospital of Chengdu Military Command, Kunming, Yunnan, China

**Keywords:** apoptosis, autophagy, necroptosis, chemotherapy resistance, osteosarcoma

## Abstract

Osteosarcoma is the most common primary bone tumor in children and adolescents. Although combined therapy including surgery and multi-agent chemotherapy have resulted in great improvements in the overall survival of patients, chemoresistance remains an obstacle for the treatment of osteosarcoma. Molecular targets or effective agents that are actively involved in cell death including apoptosis, autophagy and necroptosis have been studied. We summarized how these agents (novel compounds, miRNAs, or proteins) regulate apoptotic, autophagic and necroptotic pathways; and discussed the current knowledge on the role of these new agents in chemotherapy resistance in osteosarcoma.

## INTRODUCTION

Osteosarcoma is one of the most common primary malignant bone diseases that severely threatened the health of children and adolescents [1]. It has very high propensity for local invasion and early systemic metastases such as lung metastasis [2, 3]. Furthermore, it is a malignant tumor in connective tissues in the human skeletal system, which has a high incidence only after multiple myeloma (MM). Osteosarcoma is derived from mesenchymal stem cells with osteogenic potential, and is characterized for its immature bone or osteoid tissue through proliferating tumor cells. Its incidence is predominantly higher in children and adolescents, with the rapid growth of bones, and individuals aged between 10 to 25 years old; and accounts for 70% of all osteosarcomas [4]. The most common complaint of osteosarcoma patients is short term pain and swelling. Usually, when patients are diagnosed with osteosarcoma, the symptoms have already lasted for several months (3-4 months, or even more than six months). If diagnosis is delayed, the surface of the skin in the tumor location becomes tight and the superficial venous filling will be obviously apparent due to tumor swelling. Pathologic fracture frequently occurs after open biopsy surgery [5]. Osteosarcoma can occur in any bone and in the metaphyseal portion of the long bone were major sites of the lesion is located, with predilection for the femur and tibia; followed by the humerus, pelvis, jaw, fibula and ribs. The knee joint is the most commonly affected site, accounting 50% of all the involved sites [6].

Osteosarcoma has a highly invasive and distant metastatic potential. It is prone to hematogenous metastasis at early onset and after surgery, which most commonly occurs in the lungs, followed by other body tissues and organs [7, 8]. Osteosarcoma has high malignancy and poor prognosis. According to statistics, approximately 85% of patients with osteosarcoma have metastasis [9]. Clinically, osteosarcoma is often treated with a combination of treatments that including surgery, chemotherapy and radiation therapy [10, 11]. Two-year survival rate reaches 15%-20%. Frequently, most patients with high-grade tumors receive approximately three months of adjuvant chemotherapy before surgery such as cisplatin, doxorubicin and methotrexate. Recently, with the application of high-dose combination chemotherapy, the five-year survival rate of patients without metastasis has increased to up to 55%-70%. However, five-year survival rate in metastatic patients was merely 5%-20% [12, 13]. Although adjuvant chemotherapy has significantly improved the survival of osteosarcoma patients, some serious problems continue to exist, including severe side effects, and relapse or metastatic disease [14]. Thus, determining how to overcome chemotherapy drug resistance is important in the treatment of osteosarcoma. Therefore, it is urgent and important to clarify the mechanism of its pathogenesis, and determine an effective method to treat osteosarcoma.

## PROGRAMMED CELL DEATH IN THE TREATMENT OF OSTEOSARCOMA

Chemoresistance is an obstacle in the treatment of osteosarcoma. Thus, determining a method on how to efficiently induce cell death and overcome chemoresistance is important in the treatment of human osteosarcoma. Cell death in its various forms play an important role during development, injury and cancer [15, 16]; which mainly includes programmed cell death and non-programmed cell death [17, 18]. Due to limited knowledge, programmed cell death has been frequently referred to cell apoptosis for a long period of time. In recent years, programmed cell death has been expanded to include autophagy and necroptosis; both of which play an equally important role in apoptosis in the development of organisms [19–21]. At present, programmed cell death has included cell apoptosis, autophagy and necroptosis (programmed necrosis) [22]. In the present study, the role of apoptosis, autophagy and necroptosis on the development and progression of human osteosarcoma has been summarized. Future challenges were also highlighted and unsolved questions related to these topics were proposed.

### Cell apoptosis and osteosarcoma

Apoptosis has been traditionally thought to be an active form of cell death, and apoptotic signaling pathways have been clearly elucidated in different kinds of tumor cells. A brief introduction of the apoptotic signaling pathway was first presented, and effective agents that induce the apoptosis of osteosarcoma cells in cancer therapy were summarized.

#### Apoptotic signaling pathway

Apoptosis is normally recognized as programmed self-destruction or suicide, which is characterized by stereotypical morphological changes that include cell shrinkage and deformation, dynamic membrane blebbing, chromatin condensation, vacuolization of the mitochondria, and detachment from neighboring cells [23, 24]. An apoptotic cell is gradually fragmented into apoptotic bodies and engulfed by macrophages without inducing inflammatory responses [25, 26]. To date, 14 mammalian caspases have been reported, which are mainly divided into three groups: apoptotic initiator caspases, apoptotic effector caspases and inflammatory caspases [27, 28].

**Figure 1:**
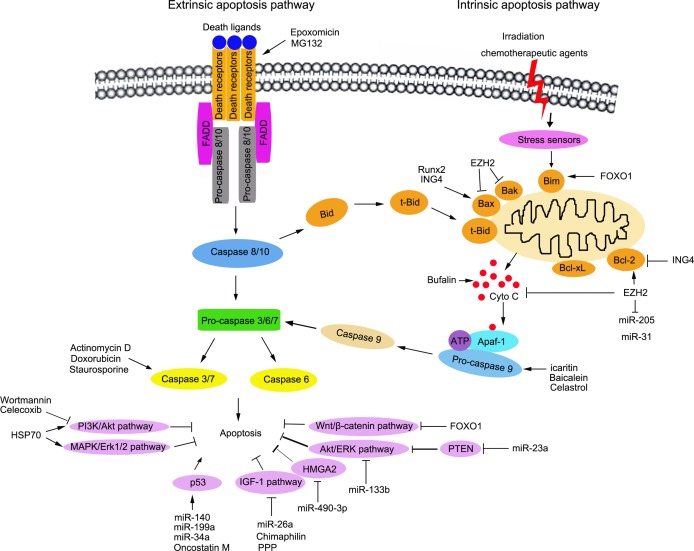
Schematic representation of the signalling pathways to apoptosis and the regulation of apoptosis in osteosarcoma therapy The two major apoptosis signaling pathways to induce apoptotic cell death are shown: extrinsic (death receptor mediated) apoptosis pathways and intrinsic (mitochondrial mediated) apoptosis pathways. In extrinsic pathway, cell apoptosis is initiated by binding of death ligands and death receptors. Next, the death-inducing siganalling complex (DISC) is assembled and caspase-8/10 is activated. In intrinsic apoptosis pathway, cell apoptosis is induced by a variety of intracellular stimuli, including damages, growth factor deprivation or oxidative stress. The cytochrome C is released from the mitochondria resulting in the formation of the apoptosome and activation of caspase-9. The activated caspase-8 and 9 trigger the execution of apoptosis by activating the downstream caspase-3, 6 and 7. It has been reported in the literature, the agents, such as natural compounds, miRNA and proteins are effective to promote or block cell apoptosis in treatment of osteosarcoma.

Furthermore, there are two major apoptosis signaling pathways that induce apoptotic cell death: extrinsic apoptosis pathways and intrinsic apoptosis pathways. The extrinsic apoptosis pathway is initiated by the binding of death ligands and death receptors. Death receptors belong to the tumor necrosis factor receptor superfamily, including TNF-R1 (DR1/CD120a/p55/p60), Fas (Apo-1/CD95), DR3 (APO-3/LARD/TRAMP), TRAIL-R1 (DR4/APO-2), TRAIL-2 (DR5), DR6, ectodysplasin A receptor (EDAR), and nerve growth factor (NGFR) [29–32]. Then, the death-inducing signaling complex (DISC) is assembled, including the Fas-associated death domain-containing protein (FADD) and the initiator caspases, procaspase-8/10 [33, 34]. In the complex, the interaction between FADD and caspase-8/10 mediates the close proximity and dimerization of the two caspase-8 molecules, resulting in autoproteolytic cleavage and the activation of caspase-8/10 [35]. The activated caspase-8/10 cleaves and activates the effector caspases (caspase-3, -6 and -7), which subsequently target a range of cellular substrates that lead to apoptosis [36]. The intrinsic apoptotic pathway is induced by a variety of intracellular stimuli such as damages mediated by irradiation or chemotherapeutic agents, growth factor deprivation, or oxidative stress [37, 38]. In this pathway, the disruption of the mitochondrial membrane leads to the release of pro-apoptotic proteins from the mitochondrial intermembrane space into the cytoplasm, which relies on the apoptosome including procaspase-9, apoptotic protease activating factor 1 (Apaf-1) and cytochrome c [38–41]. The apoptosome leads to the aggregation and activation of caspase-9, and the activated caspase-9 subsequently cleaves and activates the downstream effector caspases (caspase-3, 6 and 7) to execute cell death [42]. Proteins in the Bcl-2 family regulate the intrinsic apoptotic pathway. To date, 20 members have been found in the Bcl-2 family; which is divided into three subfamilies, depending on the presence of conserved Bcl-2 homology (BH) domains[43, 44]. One subfamily contains anti-apoptotic proteins including Bcl-2, Bcl-XL, Bcl-w and Mcl-1 [45, 46]. The other two subfamilies comprise of pro-apoptotic proteins such as Bak, Bax and Bok [47, 48]. Importantly, cross-talks exist between these extrinsic and intrinsic apoptosis signaling pathways; and each play a positive role in amplifying the apoptosis cascade (Figure 1).

#### The apoptosis of osteosarcoma cells in cancer therapy

A successful cancer therapy requires the selective distruction of osteosarcoma cancer cells. It is important to induce cell apoptosis and sensitize resistant osteosarcoma cells in osteosarcoma therapies [49, 50]. Recently, new agents have been gradually reported by different groups of researchers. Despite the improved prognosis, resistance to chemotherapy remains an obstacle in the treatment of osteosarcoma [51]. The identification of signals and effective agents that promote cell death may provide clues for developing new therapeutic strategies for chemoresistant osteosarcoma [52, 53]. Effective agents such as natural compounds, miRNA and proteins in treatment of osteosarcoma were summarized.

### MiRNAs regulate apoptosis in osteosarcoma

Numerous factors have been reported to play important roles in the regulation of apoptotic pathways such as p53, p21, insulin-like growth factor 1(IGF-1), HMGA2, PTEN and AKT. Many miRNAs have been reported to be involved in regulating the apoptosis pathway by targeting or regulating key factors in the chemoresistance of osteosarcoma cells [14]. The ectopic overexpression of miR-140 increased the expression of both p53 and p21 proteins, and contributed in increasing chemosensitivity to MTX and 5-FU treatment for human osteosarcoma cells [54]. Moreover, miR-199a and miR-34a induced the apoptosis of human osteosarcoma cells *via* the p53 signaling pathway [55]. MiR-26a exert tumor-suppressor function by the direct targeting of insulin-like growth factor 1 (IGF-1) and inhibiting IGF-1 expression in osteosarcoma. The ectopic expression of miR-490-3p decreased cell proliferation, induced apoptosis in osteosarcoma cells, and inhibited tumorigenicity in a mouse xenograft model. The mechanism was that miR-490-3p bound directly to HMGA2 mRNA 3′UTR and decreased HMGA2 levels [56]. MiR-133b was downregulated in human osteosarcoma, and the overexpression of miR-133b in osteosarcoma cell lines U2-OS and MG-63 inhibited cell proliferation, invasion and migration, and induced apoptosis. This may function as a tumor suppressor gene in osteosarcoma by decreasing the expression of predicted target genes BCL2L2, MCL-1, IGF1R and MET, as well as the expression of phospho-Akt and FAK [57]. In human osteosarcoma cell lines MG63, HOS58 and SaoS-2, miR-23a specifically targeted the 3′-untranslational region of PTEN and negatively regulated the expression of PTEN; while miR-23a-mediated the suppression of PTEN, which led to the activation of the AKT/ERK pathways and enhanced migration and invasion in osteosarcoma cells [58].

### The compounds that regulate cell apoptosis in osteosarcoma

A phenotypic high-throughput screening campaign was performed in a 25,000-small-molecule diversity library. Two compounds (doxorubicin and staurosporine) were found to selectively target osteosarcoma cells, which could induce caspase 3 and 7 activity in the U2OS cell line and promote cell apoptosis in osteosarcoma cell lines [59]. Chimaphilin, an active compound separated from pyrola, can inhibit proliferation and induce apoptosis in multidrug resistant osteosarcoma cell lines through insulin-like growth factor-I receptor (IGF-IR) signaling, as well as increase the sensitivity of doxorubicin in doxorubicin-resistant osteosarcoma cell lines [60]. Claritin, a prenylflavonoid derivative of the Chinese tonic herb Epimedium, could suppress proliferation in human osteosarcoma cells *in vitro* by upregulating caspase-3 and caspase-9 expression and increasing the level of cleaved caspase-3 [61]. Tanshinone IIA (Tan IIA) is an active ingredient extracted from the widely used Danshen root (Salvia miltiorrhiza Bunge), which induces apoptosis and inhibits the proliferation and invasion of osteosarcoma MG-63 cells by caspase activation [62]. Celastrol is an active compound extracted from the root bark of Tripterygium wilfordii Hook F, which induce apoptosis in human osteosarcoma cells *via* the mitochondrial apoptotic pathway, and result in caspase-3 and -9 activation and PARP cleavage [63]. Additionally, a homogeneous polysaccharide (TRP) was isolated and purified from Trametes robiniophila Murrill, which could induce apoptosis through the intrinsic mitochondrial pathway in human osteosarcoma (U-2 OS) cells [64]. Furthermore, bufalin induced apoptosis in the U2OS human osteosarcoma cell line, which was accompanied with a significant reduction in mitochondrial membrane potential, the release of mitochondrial cytochrome c into the cytosol, the activation of caspase-3, caspase-9 and poly (adenosine diphosphate ribose) polymerase, as well as the downregulation of B-cell lymphoma 2 (Bcl-2)/Bcl-2-associated X protein; suggesting that bufalin induced apoptosis by triggering the mitochondrial pathway [65]. Baicalein is a new drug, and baicalein-induced apoptosis in osteosarcoma cells was *via* a mitochondrial pathway that involved both caspase-dependent and -independent mechanisms. However, baicalein treatment notably upregulated the expression of HSP70, which partially prevented human osteosarcoma cells from undergoing apoptosis, and decreased the sensitivity of osteosarcoma cells to baicalein *via* the activation of the PI3K/AKT and MAPK/ERK pathways [66]. Celecoxib, a cyclooxygenase-2 inhibitor, induced apoptosis in human osteosarcoma cell line MG-63 *via* the downregulation of PI3K/Akt, and decreased the level of survival and bcl-2 in cells treated with the combination of celecoxib and cisplatin or wortmannin, a specific PI3K inhibitor [67]. Cyclolignan picropodophyllin (PPP), an insulin-like growth factor-I receptor tyrosine kinase inhibitor, inhibited proliferation and induced apoptosis in multidrug resistant osteosarcoma cell lines by monitoring poly (ADP-ribose) polymerase and its cleavage product [68]. Epoxomicin, a proteasome inhibitor, sensitized resistant osteosarcoma cells to TRAIL-induced apoptosis in two TRAIL-resistant OS cell lines, Saos-2 and MG-63; and significantly increased caspase-3, caspase-8, caspase-9 activities and Bax protein levels [69]. Additionally, MG132 (proteasome inhibitor) enhanced TRAIL-induced apoptosis and inhibited the invasion of human osteosarcoma OS732 cells [70].

### Protein factors regulate cell apoptosis in osteosarcoma

Some protein factors have been reported to regulate apoptosis-related proteins or caspases, and contributed to cell apoptosis in osteosarcoma. Overexpressed inhibitor of growth 4 (ING4) increased the mRNA levels of p21, Bax and caspase-3, and decreased the ratio of Bcl-2/Bax; which induced the apoptosis of osteosarcoma cells by the activation of the mitochondria pathway and blockage of the NF-κB signaling pathway [71]. Oncostatin M (OSM), a cytokine of the interleukin-6 family, sensitized and transformed osteoblasts to apoptosis by a mechanism that implicated the activation and nuclear translocation of STAT5 and p53, and an increased Bax/Bcl-2 ratio [72]. The Runx family of transcription factors regulates cell growth and differentiation. Runx2 acts as a proapoptotic factor in osteosarcoma cells by the direct targeting of Bax [73]. Moreover, loss of Runx2 sensitized osteosarcoma to chemotherapy-induced apoptosis [74]. FOXO transcription factors, especially FOXO1, has a tumor-suppressing role in osteasarcoma cells, partially by suppression of the Wnt/β-catenin pathway [75]. Actinomycin D (ActD), a well known transcription inhibitor, inhibited cell proliferation and promoted apoptosis in osteosarcoma cells by increasing the levels of cleaved caspase-3 with increasing ActD concentrations and treatment times [76]. Enhancer of zeste homolog 2 (EZH2) is the catalytic subunit of polycomb repressive complex 2. The knockdown of EZH2 caused a decrease in anti-apoptotic Bcl-2 and an increase in pro-apoptotic Bax and Bak proteins, as well as an increase in the expression of cytochrome C in the cytosol; indicating that EZH2 suppressed cell apoptosis through the intrinsic apoptotic pathway [77]. Additionally, EZH2 could inhibit cell apoptosis by coordinating the epigenetic silencing of two proapoptotic microRNAs (miRNA), miR-205 and miR-31 [78].

### Autophagy and treatment of osteosarcoma

The treatment of osteosarcoma usually requires the combination of surgical resection and systemic chemotherapy. However, the chemoresistance of osteosarcoma remains an obstacle in clinical therapy [79–81]. Overcoming chemoresistance is one approach to improve survival in osteosarcoma patients. To date, the role of autophagy has not been clearly elucidated in promoting chemosensitivity or chemoresistance in osteosarcoma [82]. The role of autophagy is not constant in osteosarcoma therapies. In some circumstances, autophagy can promote cell survival; and in other circumstances, it contributes to cell death. Moreover, autophagy could promote both chemosensitivity and chemoresistance in different circumstances during osteosarcoma therapy.

#### Signaling pathways to autophagy

Autophagy is a highly conservative process characterized by the vesicular sequestration and degradation of cytoplasmic components [83]. Autophagy is trigged and precisely regulated by nutrient deprivation, growth factors, hormones, intracellular energy information and cell stressors such as hypoxia, osmotic stress, reactive oxygen species (ROS) and viral infection, etc. [84]. There are several main steps in autophagy: induction, vesicle nucleation and elongation, autophagosome formation and autolysosome formation [85]. To date, more than 30 autophagy- related genes (Atg) have been found to be involved, and several molecular complexes are essential in the process of autophagy [86, 87]. These are mainly divided into five groups according to its function in the process of autophagy: Atg1 protein kinase complex, class III phosphatidylinositol 3 kinase (PI3K)-Beclin 1 complex, Atg12 conjugation system, Atg8 conjugation system, and Atg9 [88].

The Atg1 protein kinase complex is essential for the induction of autophagy. Unc-51-like kinase 1 (ULK1) and −2(ULK-2) are two mammalian homologs of Atg1. ULK1, mammalian Atg13 (mAtg13), focal adhesion kinase family interacting protein of 200kDa (FIP200), and Atg101 forms the ULK1 complex [89, 90]. The mammalian target of the rapamycin complex (mTORC) binds to and inactivates ULK1/2 and Atg13. Upon mTOR inhibition, ULK1 and ULK2 are activated, and subsequently followed by phosphorylate FIP200 and ATG13; which initiates autophagy activity [91]. The PI3K complex, which is essential for vesicle nucleation, is composed of p150, PI3 kinase class III, Beclin-1 and ATG14; which are regulated by several factors including Bcl-2, Bcl-XL and the Run domain protein Rubicon and Ambra 1 [91]. The conjugation of Atg12 and Atg8 is essential for the formation of autophagosomes. In the Atg12 conjugation system, Atg12 is activated by Atg7 and Atg10, and conjugated to Atg5; which promotes the formation of the autophagy precursor [92, 93]. Atg5 interacts further with autophagy-related 16-like1 (ATG16L1) to form the ATG16L1-ATG5-ATG12 complex [94]. Meanwhile, in the Atg8 conjugation system, Atg8 is initially activated by Atg4. The activated Atg8 bonds with E1-like enzyme, Atg7; and is transferred to the E2-like enzyme, Atg3. Finally, Atg8 is conjugated to PE to form the Atg8-PE complex, which exists in a tight membrane-associated form [95, 96]. The conjugation of Atg12-Atg5 facilitates the protein lipidation of Atg8 and Atg8, and phosphatidylethanolamine supports membrane expansion as a scaffold protein in the formation of autophagosomes [97].

**Figure 2:**
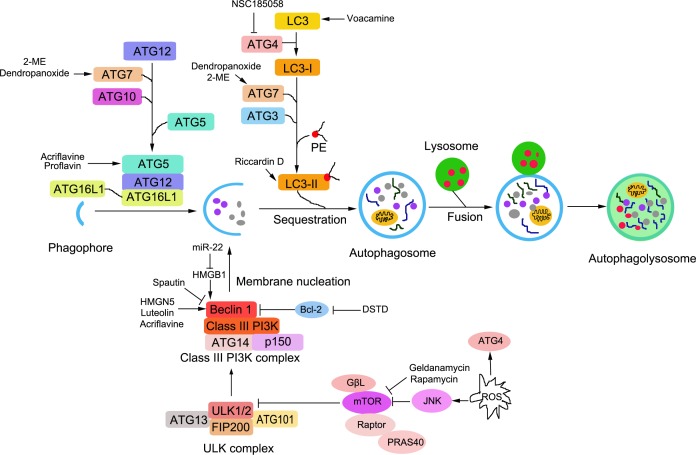
Schematic representation of signalling pathways to autophagy and the regulation of autophagy in osteosarcoma cells The steps of autophagy are including induction, vesicle nucleation, vesicle elongation, autophagosome formation, fusion and autolysosome formation. Autophagy is trigged and precisely regulated by nutrient deprivation, growth factors, hormones, intracellular energy information and cell stressors, such as hypoxia, osmotic stress, reactive oxygen species (ROS) and viral infection, etc. The autophagy- related genes (Atg) are involved in several molecular complexes and the major 5 groups are essential in the process of autophagy including: Atg1 protein kinase complex, the class III phosphatidylinositol 3 kinase(PI3K)-Beclin 1 complex, Atg12 conjugation system, Atg8 conjugation system and Atg9. Chemoresistance of osteosarcoma is still an obstacle in clinical therapy and some effective agents to induce autophagy are listed in the therapy for osteosarcoma.

Briefly, the Atg1 protein kinase complex (the ULK complex) is essential for the induction of autophagy. The class III PI3 kinase-Becline complex is essential for vesicle nucleation, and is crucial for recruiting the Atg12-Atg5 conjunction to the pre-autophagosomal structure. Atg12-Atg5 conjugation is important for vesicle elongation and for the proper localization of Atg8. ATG9 have been thought to be involved in the delivery of membrane lipids to form autophagosomes. Major autophagy cascades are depicted in Figure 2.

#### Autophagy and osteosarcoma therapy

#### Autophagy promotes chemosensitivity in osteosarcoma

Flavonoid luteolin enhanced doxorubicin-induced autophagy in human osteosarcoma U2OS cells through upregulating beclin1, which revealed a synergistic cytotoxicity, leading to U2OS cell death [98]. The 3,4-dihydroxy-9,10-secoandrosta-1,3,5,7-tetraene-9,17-dione (DSTD), a novel androstenedione derivative, inhibited macrophage migration inhibitory factor (MIF) expression in MG-63 and U2OS cells. The inhibition of MIF by DSTD promoted autophagy by inducing Bcl-2 downregulation and the translocation of the high mobility group box 1 protein (HMGB1); suggesting that in the presence of chemotherapy drugs, DSTD enhanced chemosensitivity by decreasing HMGB1 levels and overcoming drug resistance in osteosarcoma [99]. Moreover, two conjugation systems are also regulated in autophagy. The bisindolic alkaloid voacamine (VOA), isolated from the plant Peschiera fuchsiaefolia, is an autophagy inducer that can exert an apoptosis-independent cytotoxic effect on both wild-type and MDR tumor cells by promoting LC3 expression and conversion [100]. The 2-Methoxyestradiol (2-ME) treatment was correlated with the formation of autophagosomes in human osteosarcoma cells by inducing the conversion of microtubule-associated protein LC3-I to LC3-II, requiring ATG7 expression [101]. Moreover, autophagy could play an important role in 2-ME-mediated anti-tumor actions in osteosarcoma.

#### Autophagy promotes chemoresistance in osteosarcoma

Autophagy is initiated by autophagosome formation upon mammalian target of rapamycin inhibition and phosphatidylinositol 3-phosphate [PtdIns(3)P] generation, and the PI3K complex is essential for vesicle nucleation. In osteosarcoma cells, HMGB1 levels were upregulated and HMGB1-mediated autophagy contributed to chemotherapy resistance. However, possibly as a compensatory effect, miR-22 was also upregulated during chemotherapy; and overexpressed miR-22 targeted the 3′UTR of HMGB1 and inhibited HMGB1-promoted autophagy [102]. Additionally, miR-22 inhibited osteosarcoma cell proliferation and migration by targeting HMGB1 and inhibiting HMGB1-mediated autophagy [103]. The upregulated HMGB1 increased the formation of the Beclin1-PtdIns3KC3 complex and stimulated autophagosome maturation and autophagy by competing with Bcl-2 to bind Beclin1. The inhibition of both HMGB1 and autophagy increased the drug sensitivity of osteosarcoma cells *in vivo* and *in vitro*. Furthermore, the ULK1-FIP200 complex was required for the interaction between HMGB1 and Beclin1, which promoted Beclin1-PtdIns3KC3 complex formation during autophagy; suggesting that HMGB1-mediated autophagy contributed to osteosarcoma drug resistance [104]. Additionally, high-mobility group nucleosome-binding domain 5 (HMGN5) was highly expressed in osteosarcoma tumors, especially in post-treatment tumors. The overexpression of HMGN5 reduced the chemosensitivity of osteosarcoma cells *in vitro*, and the mechanistic investigation revealed that HMGN5 increased drug resistance by upregulating autophagy accompanied by increasing levels of ULK1, Beclin1 and LC3-ii/I [105]. MiR-101 decreased the levels of Atg4, a regulator of two conjugation systems, which inhibited Dox-promoted autophagic vesicle formation in U-2 OS cells, and blocked autophagy by miR-101 sensitized osteosarcoma cells to chemotherapy [106].

#### Interplay between autophagy and apoptosis

The precise role of autophagy in the development and progression of osteosarcoma, as well as the response to clinical therapy, remains unclear. Reports have suggested that autophagy can promote both cell survival and cell death [107]. In some cellular circumstances, autophagy is involved in the cell survival pathway to suppress apoptosis; while in other circumstances, it can lead to cell death itself, either in collaboration with apoptosis or as a back-up mechanism when the former is defective [108]. There is a close relationship between autophagy and apoptosis during cell death [109] such as regulators PI3K and Akt, which play an important role in the cross-talk between apoptosis and autophagy. It is possible that either cell survival or death may be regulated by the selective autophagic clearance of the cytoplasmic material [110]. Therefore, a better understanding of the regulation of autophagy in humans and a combinatorial approach that utilizes autophagy modulators would help provide better targets for osteosarcoma therapies (Table 1).

**Table 1:** The interplay between autophagy and apoptosis in osteosarcoma

Agent	Description	Interplay between autophagy and apoptosis
The antagonistic relationship between autophagy and apoptosis		
Beclin-1	An autophagy gene	Knockdown of Atg 6, Beclin-1 decreased cell growth, invasion, and metastasis [115].
Geldanamycin (GA)	Hsp90 inhibitor	GA induced autophagy by inhibiting Akt/mTOR/p70S6K signaling and induced apoptosis in the caspase-dependent apoptotic pathway in KTHOS cells. However, the combinated treatment of GA and the autophagy inhibitor 3-methyl-adenine (3-MA) enhanced GA-induced apoptosis in KTHOS cells [116].
NSC185058	A ATG4B antagonist	NSC185058 effectively inhibited ATG4B activity in vitro and in cells while having no effect on MTOR and PtdIns3K activities. ATG4B antagonist had a negative impact on the development of Saos-2 osteosarcoma tumors *in vivo* [117].
Dendropanoxide (DP)	A compound	DP induced autophagy through ERK1/2 activation in MG-63 human osteosarcoma cells, but inhibiting autophagy enhanced dendropanoxide-induced apoptosis [119].
Dihydroptychantol A(DHA)	a macrocyclic bisbibenzyl derivative	DHA-mediated apoptotic cell death was potentiated by the autophagy inhibitor 3-methyladenine, suggesting that autophagy may play a protective role that impedes the eventual cell death [120]
The synergistic relationship between apoptosis and autophagy		
Proflavin	An acridine derivative	Proflavin exerted anticancer potential through the synergistic activity of apoptosis and autophagy [111]
Celastrol	a triterpene	Inhibiting celastrol-induced autophagy diminished apoptosis and celastrol induced autophagy and apoptosis via the ROS/JNK signaling pathway in human osteosarcoma cells [112]
Acriflavine	an acridine derivative	Acriflavine-induced cell death was attributed to both apoptosis and autophagy. The antiseptic agent, acriflavine, has anticancer potential through synergistic activity of apoptosis and autophagy [113].
Riccardin D	A macrocyclic bisbibenzyl compound	Riccardin D induces cell death by activation of apoptosis and autophagy in osteosarcoma cells [114]

#### Apoptosis and autophagy has a synergistic activity

Proflavin was one of the novel acridine derivatives that inhibited the proliferation of MG63 cells in a dose-dependent manner, and exerted anti-cancer potential through the synergistic activity of apoptosis and autophagy [111]. Celastrol, a triterpene from traditional Chinese medicine, has been proven to possess potent anti-tumor effects on various cancers. Celastrol induced apoptosis and autophagy *via* the ROS/JNK signaling pathway in human osteosarcoma cells, and the suppression of autophagy diminished apoptosis [112]. Acriflavine suppressed the growth of human osteosarcoma cells through apoptosis and autophagy, and both contributed to cell death [113]. Riccardin D-induced autophagy was accompanied by the accumulation of LC3B-II and the formation of AVOs and punctate dots. Moreover, riccardin D induced cell death through the activation of apoptosis and autophagy in osteosarcoma cells [114].

#### Antagonistic relationship between autophagy and apoptosis

Beclin-1, a well-known key regulator of autophagy, has been implicated in many disorders including cancer, aging and degenerative diseases. The knockdown of autophagy-related protein 6 (Atg 6), Beclin-1, decreased cell growth, invasion and metastasis; suggesting that the inhibition autophagy had a positive effect on chemotherapy-induced cytotoxicity in osteosarcoma cells and increased the efficacy of anti-cancer agent therapy [115]. Hsp90 inhibitor, geldanamycin (GA) induced autophagy in KTHOS cells by inhibiting Akt/mTOR/p70S6K signaling. It also induced the caspase-dependent apoptotic pathway in KTHOS cells. Importantly, the combined treatment of GA and autophagy inhibitor 3-methyl-adenine (3-MA) enhanced GA-induced apoptosis in KTHOS cells [116]. NSC185058, a novel ATG4B antagonist, is a small compound from the NCI library, which bond to the active site of ATG4B. NSC185058 could effectively inhibit ATG4B activity *in vitro* and in cells, while having no effect on MTOR and PtdIns3K activities; suggesting that NSC185058 is effective in suppressing autophagy *in vivo* and in attenuating osteosarcoma tumor growth [117]. Rapamycin inhibited cell proliferation and decreased the phosphorylation of mTOR pathway components in MG63 cells, while Spautin-1 suppressed the protective mechanism induced by rapamycin in osteosarcoma cells and effectively induced apoptosis [118]. Dendropanoxide (DP) newly isolated from leaves and stem of Dendropanax morbifera Leveille could induce autophagy through ERK1/2 activation in MG-63 human osteosarcoma cells, but inhibiting autophagy enhanced dendropanoxide-induced apoptosis [119]. Dihydroptychantol A, a macrocyclic bisbibenzyl derivative, induced autophagy following apoptosis associated with the p53 pathway in human osteosarcoma U2OS cells. DHA-mediated apoptotic cell death was potentiated by the autophagy inhibitor 3-methyladenine, suggesting that autophagy may play a protective role that impedes eventual cell death [120]. Osteosarcoma treatments often fail due to the development of chemoresistance to apoptosis, and PERK activates osteosarcomatous autophagy *via* inhibiting the mTORC1 pathway. In order to explore the relationship of autophagy and apoptosis in osteosarcomatous resistance, the knockdown of PERK was induced by using RNAi in osteosarcoma, which suppressed autophagy; and this resulted in osteosarcomatous apoptosis [121].

### Necroptosis

Necrosis is traditionally thought to be a passive and unprogrammed cell death, which is caused by factors including infection, toxins, or trauma; and is characterized by swelling of the organelles, plasma membrane permeabilization, cellular collapse, mitochondria dysfunction, release of cellular contents, and inflammation in the surrounding tissues [122, 123]. However, emerging evidence has shown that necrosis can be induced and regulated in a similar manner to apoptosis. This has been recently identified as a form of programmed cell death that is different with traditional necrosis and apoptosis [124, 125]. Furthermore, it is a regulated cell death with the morphological characterization of necrosis; which can be pharmacologically inhibited by certain chemical compounds such as necrostatin-1 [126]. Recently, it has been found that the necroptosis signaling pathway has also contributed to the treatment of osteosarcoma.

#### Signaling pathways to necroptosis

The signaling pathway of necroptosis has gradually been clarified recently. This is triggered by multiple stimulators, and interacts with death receptors (TNFR1, TRAIL-R or Fas), T-cell receptors (TCR), Toll-like receptors (TLRs), cellular metabolic and genotoxic stress, and a number of anti-cancer agents [127–129]. In TNF-α induced necroptosis, three complexes are essential and are key regulators that trigger this response, including complex I, complex IIa and complex IIb [130, 131].

**Figure 3:**
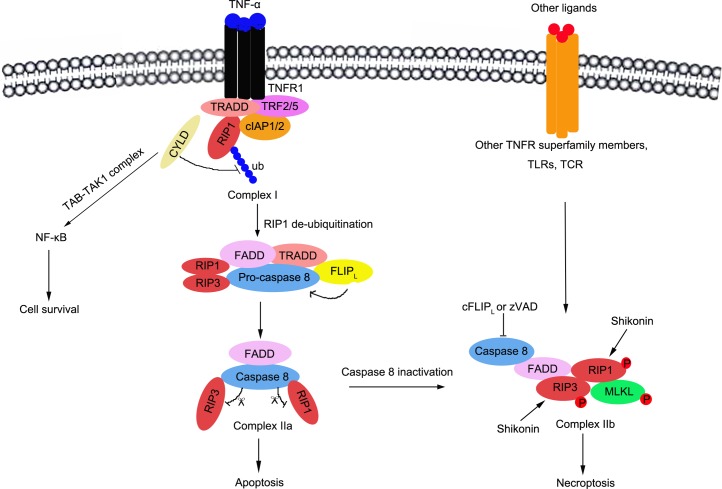
Schematic representation of signalling pathways to necroptosis and the regulation of necroptosis in osteosarcoma The necroptosis is triggered by multiple stimulators, including death receptor (TNFR1, TRAIL-R or Fas), T-cell receptor (TCR), Toll-like receptors (TLRs), cellular metabolic and genotoxic stresses, and a lot of anti-cancer agents, etc. In TNF-α induced necroptosis, three complexes are essential including complex I(TNFR1-associated death domain protein (TRADD), TNF receptor-associated factor 2(TRAF2), RIP1, cellular inhibitor of apoptosis protin1/2(cIAP1/2), and the linear ubiquitin chain assembly complex (LUBAC)), complex IIa(RIP1, TRADD, FADD(FAS-associated death domain), RIP3, pro-caspase8 and FLICE inhibitory proteins (FLIP)) and complex IIb(Caspase-8, FADD, RIP1, RIP3 and mixed lineage kinase domain-like (MLKL) protein).

The interaction of TNF-α and TNFR1/2 leads to the formation of the intracellular complex at the cytoplasmic membrane (complex I), which includes TNFR1-associated death domain protein (TRADD), TNF receptor-associated factor 2(TRAF2), RIP1, cellular inhibitor of apoptosis protin1/2 (cIAP1/2), and the linear ubiquitin chain assembly complex (LUBAC) [132]. Within complex I, RIP1 is ubiquitinated by cIAP1/2; and K63-ubiquitinated RIP1 serves as scaffolds, which promotes to recruit and activate the TAB-TAK1 and the IKKα-IKKβ-NEMO complexes, leading to NF-κB activation, the upregulation of pro-survival genes, and the inhibition of cell death[133, 134]. In contrast, K63-ubiquitinated RIP1 is deubiquitinated by cylindromatosis (CYLD), and the released RIP1 constitutes to form complex IIa including RIP1, TRADD, FADD (FAS-associated death domain), RIP3, pro-caspase-8 and FLICE inhibitory proteins (FLIP) [135]. In complex IIa, caspase-8 is released and activated by FLIP; and the activated caspase-8 cleaves RIP1 and RIP3, which prevents their trans-phosphorylation and the activation of complex IIb [136]. Thus, it successfully initiates the pro-apoptotic caspase activation cascade, which leads to cell apoptosis. However, when caspase-8 is deleted or inactivated by pharmacological inhibitors such as Z-VAD, it loses the ability to cleave RIP1 and RIP3 [131, 137]. The complex IIb would be formed including caspase-8, FADD, RIP1, RIP3 and mixed lineage kinase domain-like (MLKL) protein[138]. Then, the activated RIP1 and RIP3 trans-phosphorylates each other and initiates necroptosis [139]. Both RIP1 and RIP3 necrosome recruits and activates MLKL and phosphoglycerate mutase 5(PGAM5). MLKL is phosphorylated and multimerized, is inserted into the plasma membrane, and forms channels that increases Na+ influx, osmotic pressure and membrane rupture; ending with necroptosis induced-cell death [129, 136]. PGAM5 recruits and activates mitochondrial fission factor dynamin-related protein 1 (Drp1), leading to mitochondrial fission; which is a key factor for necrosis execution [137, 140] (Figure 3).

#### Necroptosis and osteosarcoma therapy

Presently, few literatures have referred on necroptosis and osteosarcoma therapy. Shikonin, an effective constituent extracted from Chinese medicinal herbs, has been demonstrated to induce necroptosis in some cancers. The anti-tumor effects of shikonin on osteosarcoma were partly due to inducing RIP1 and RIP3 dependent necroptosis; indicating that shikonin could be a potential anti-tumor agent on the treatment of primary and metastatic osteosarcoma [141]. Shikonin has dually functioned as a proteasome inhibitor and necroptosis inducer in multiple myeloma cells. Interestingly, the combination of a heat shock protein inhibitor with low dose SHK enhanced cell apoptosis, while high-dose SHK induced necroptosis in MM cells [142]. Moreover, hiporfin-mediated photodynamic therapy in the preclinical treatment of osteosarcoma demonstrated that cell death caused by hiporfin-PDT could be rescued by Nec-1, but not by Z-VAD-FMK [143]. Additionally, RIP3 expression induced death profile changes in U2OS osteosarcoma cells after 5-aminolevulic acid (5-ALA)-mediated photodynamic therapy (PDT), suggesting that autophagy was likely to play a protective role against PDT-induced cell death and allow better survival for RIP3-U2OS cells [144].

## CONCLUSION AND PERSPECTIVES

In this review, we summarized how apoptosis, autophagy and necroptosis affect the proliferation and invasion of osteosarcoma cells, as well as the potential target in apoptosis, autophagy and necroptosis for clinical therapy of drug-resistant osteosarcoma. However, many issues remain to be clarified.

To date, limited references have been reported on necroptosis in human osteosarcoma. No study has reported the role of necroptosis in the drug resistance of osteosarcoma.

Metastases in osteosarcoma patients such as lung metastases or pulmonary metastasis can be easily observed, and indicate poor prognosis. The interplay between apoptosis, autophagy, necroptosis, tumor metastases, as well as the mechanism of metastases in osteosarcoma, needs to be clearly explored.

Drug resistance is the biggest obstacle in the therapy of human osteosarcoma. The role of autophagy has not been clearly clarified in terms of promoting chemosensitivity or chemoresistance in osteosarcoma. Furthermore, the interplay between autophagy and apoptosis is complex; for example, autophagy can promote both cell survival and cell death. The potential targets in signaling pathways and effective drug candidates are needs to be further investigated.

Cell apoptosis, autophagy and necroptosis all contributed to cell death in osteosarcoma cells. The cross-talk between these different regulatory pathways needs to be clearly clarified.

## References

[1] Sampson VB, Yoo S, Kumar A, Vetter NS, Kolb EA (2015). MicroRNAs and Potential Targets in Osteosarcoma: Review. Frontiers in pediatrics.

[2] Zhao H, Yao Y, Wang Z, Lin F, Sun Y, Chen P (2010). Therapeutic effect of pirarubicin-based chemotherapy for osteosarcoma patients with lung metastasis. Journal of chemotherapy.

[3] He A, Yang X, Huang Y, Feng T, Wang Y, Sun Y, Shen Z, Yao Y (2015). CD133(+) CD44(+) Cells Mediate in the Lung Metastasis of Osteosarcoma. Journal of cellular biochemistry.

[4] Daw NC, Chou AJ, Jaffe N, Rao BN, Billups CA, Rodriguez-Galindo C, Meyers PA, Huh WW (2015). Recurrent osteosarcoma with a single pulmonary metastasis: a multi-institutional review. British journal of cancer.

[5] Friebele JC, Peck J, Pan X, Abdel-Rasoul M, Mayerson JL (2015). Osteosarcoma: A Meta-Analysis and Review of the Literature. American journal of orthopedics.

[6] Salunke AA, Chen Y, Tan JH, Chen X, Khin LW, Puhaindran ME (2014). Does a pathological fracture affect the prognosis in patients with osteosarcoma of the extremities? : a systematic review and meta-analysis. The bone & joint journal.

[7] Munajat I, Zulmi W, Norazman MZ, Wan Faisham WI (2008). Tumour volume and lung metastasis in patients with osteosarcoma. Journal of orthopaedic surgery.

[8] Maeda R, Isowa N, Onuma H, Miura H, Touge H, Kawasaki Y (2008). Appearance of lung metastasis from osteosarcoma 21 years after initial treatment. General thoracic and cardiovascular surgery.

[9] Faisham WI, Mat Saad AZ, Alsaigh LN, Nor Azman MZ, Kamarul Imran M, Biswal BM, Bhavaraju VM, Salzihan MS, Hasnan J, Ezane AM, Ariffin N, Norsarwany M, Ziyadi MG, Wan Azman WS, Halim AS, Zulmi W (2015). Prognostic factors and survival rate of osteosarcoma: A single-institution study. Asia-Pacific journal of clinical oncology.

[10] Yu W, Tang L, Lin F, Yao Y, Shen Z, Zhou X (2015). High-intensity focused ultrasound: noninvasive treatment for local unresectable recurrence of osteosarcoma. Surgical oncology.

[11] Liu Y, Xu Y, Lin N, Jiang S, Wang Y, Ye Z (2015). High-dose methotrexate (HD-MTX) used as an adjunct with other chemotherapeutics for the treatment of osteosarcoma. Cell biochemistry and biophysics.

[12] Chen J, Ma L, Wei G (2014). Comment on Fu et al. : A systematic review of p53 as a biomarker of survival in patients with osteosarcoma. Tumour biology : the journal of the International Society for Oncodevelopmental Biology and Medicine.

[13] Shin SH, Jeong HJ, Han I, Cho HS, Kim HS (2013). Osteosarcoma and chondrosarcoma of the shoulder: site-specific comparative analysis. Orthopedics.

[14] He H, Ni J, Huang J (2014). Molecular mechanisms of chemoresistance in osteosarcoma (Review). Oncology letters.

[15] Elmore S (2007). Apoptosis: a review of programmed cell death. Toxicologic pathology.

[16] Zhang X, Chen Y, Jenkins LW, Kochanek PM, Clark RS (2005). Bench-to-bedside review: Apoptosis/programmed cell death triggered by traumatic brain injury. Critical care.

[17] Kroemer G, Galluzzi L, Vandenabeele P, Abrams J, Alnemri ES, Baehrecke EH, Blagosklonny MV, El-Deiry WS, Golstein P, Green DR, Hengartner M, Knight RA, Kumar S, Lipton SA, Malorni W, Nunez G (2009). Classification of cell death: recommendations of the Nomenclature Committee on Cell Death 2009. Cell death and differentiation.

[18] Li YM (1989). [Classification and mechanisms of cell death]. Zhonghua bing li xue za zhi Chinese journal of pathology.

[19] Lalaoui N, Lindqvist LM, Sandow JJ, Ekert PG (2015). The molecular relationships between apoptosis, autophagy and necroptosis. Seminars in cell & developmental biology.

[20] Safa AR (2013). Roles of c-FLIP in Apoptosis, Necroptosis, and Autophagy. Journal of carcinogenesis & mutagenesis.

[21] Long JS, Ryan KM (2012). New frontiers in promoting tumour cell death: targeting apoptosis, necroptosis and autophagy. Oncogene.

[22] Ouyang L, Shi Z, Zhao S, Wang FT, Zhou TT, Liu B, Bao JK (2012). Programmed cell death pathways in cancer: a review of apoptosis, autophagy and programmed necrosis. Cell proliferation.

[23] Willenberg HS, Bornstein SR, Dumser T, Ehrhart-Bornstein M, Barocka A, Chrousos GP, Scherbaum WA (1998). Morphological changes in adrenals from victims of suicide in relation to altered apoptosis. Endocrine research.

[24] Deschesnes RG, Huot J, Valerie K, Landry J (2001). Involvement of p38 in apoptosis-associated membrane blebbing and nuclear condensation. Molecular biology of the cell.

[25] Nunez R, Sancho-Martinez SM, Novoa JM, Lopez-Hernandez FJ (2010). Apoptotic volume decrease as a geometric determinant for cell dismantling into apoptotic bodies. Cell death and differentiation.

[26] D'Avila H, Freire-de-Lima CG, Roque NR, Teixeira L, Barja-Fidalgo C, Silva AR, Melo RC, Dosreis GA, Castro-Faria-Neto HC, Bozza PT (2011). Host cell lipid bodies triggered by Trypanosoma cruzi infection and enhanced by the uptake of apoptotic cells are associated with prostaglandin E(2) generation and increased parasite growth. The Journal of infectious diseases.

[27] Eckhart L, Ballaun C, Uthman A, Kittel C, Stichenwirth M, Buchberger M, Fischer H, Sipos W, Tschachler E (2005). Identification and characterization of a novel mammalian caspase with proapoptotic activity. The Journal of biological chemistry.

[28] Wei W, Norton DD, Wang X, Kusiak JW (2002). Abeta 17-42 in Alzheimer's disease activates JNK and caspase-8 leading to neuronal apoptosis. Brain : a journal of neurology.

[29] Gonzalvez F, Ashkenazi A (2010). New insights into apoptosis signaling by Apo2L/TRAIL. Oncogene.

[30] Hovelmeyer N, Hao Z, Kranidioti K, Kassiotis G, Buch T, Frommer F, von Hoch L, Kramer D, Minichiello L, Kollias G, Lassmann H, Waisman A (2005). Apoptosis of oligodendrocytes via Fas and TNF-R1 is a key event in the induction of experimental autoimmune encephalomyelitis. Journal of immunology.

[31] Warat M, Sadowski T, Szliszka E, Krol W, Czuba ZP (2015). The role of selected flavonols in tumor necrosis factor-related apoptosis-inducing ligand receptor-1 (TRAIL-R1) expression on activated RAW 264. 7 macrophages. Molecules.

[32] Natoni A, MacFarlane M, Inoue S, Walewska R, Majid A, Knee D, Stover DR, Dyer MJ, Cohen GM (2007). TRAIL signals to apoptosis in chronic lymphocytic leukaemia cells primarily through TRAIL-R1 whereas cross-linked agonistic TRAIL-R2 antibodies facilitate signalling via TRAIL-R2. British journal of haematology.

[33] Olsson M, Vakifahmetoglu H, Abruzzo PM, Hogstrand K, Grandien A, Zhivotovsky B (2009). DISC-mediated activation of caspase-2 in DNA damage-induced apoptosis. Oncogene.

[34] Kischkel FC, Lawrence DA, Tinel A, LeBlanc H, Virmani A, Schow P, Gazdar A, Blenis J, Arnott D, Ashkenazi A (2001). Death receptor recruitment of endogenous caspase-10 and apoptosis initiation in the absence of caspase-8. The Journal of biological chemistry.

[35] Fulda S, Meyer E, Friesen C, Susin SA, Kroemer G, Debatin KM (2001). Cell type specific involvement of death receptor and mitochondrial pathways in drug-induced apoptosis. Oncogene.

[36] Chang DW, Xing Z, Pan Y, Algeciras-Schimnich A, Barnhart BC, Yaish-Ohad S, Peter ME, Yang X (2002). c-FLIP(L) is a dual function regulator for caspase-8 activation and CD95-mediated apoptosis. The EMBO journal.

[37] Argun M, Tok L, Uguz AC, Celik O, Tok OY, Naziroglu M (2014). Melatonin and amfenac modulate calcium entry, apoptosis, and oxidative stress in ARPE-19 cell culture exposed to blue light irradiation (405 nm). Eye.

[38] Katoh I, Sato S, Fukunishi N, Yoshida H, Imai T, Kurata S (2008). Apaf-1-deficient fog mouse cell apoptosis involves hypo-polarization of the mitochondrial inner membrane, ATP depletion and citrate accumulation. Cell research.

[39] Caroppi P, Sinibaldi F, Fiorucci L, Santucci R (2009). Apoptosis and human diseases: mitochondrion damage and lethal role of released cytochrome C as proapoptotic protein. Current medicinal chemistry.

[40] Schamberger CJ, Gerner C, Cerni C (2005). Caspase-9 plays a marginal role in serum starvation-induced apoptosis. Experimental cell research.

[41] Sanchis D, Mayorga M, Ballester M, Comella JX (2003). Lack of Apaf-1 expression confers resistance to cytochrome c-driven apoptosis in cardiomyocytes. Cell death and differentiation.

[42] Arbab IA, Abdul AB, Sukari MA, Abdullah R, Syam S, Kamalidehghan B, Ibrahim MY, Taha MM, Abdelwahab SI, Ali HM, Mohan S (2013). Dentatin isolated from Clausena excavata induces apoptosis in MCF-7 cells through the intrinsic pathway with involvement of NF-kappaB signalling and G0/G1 cell cycle arrest: a bioassay-guided approach. Journal of ethnopharmacology.

[43] Zhang H, Xu Q, Krajewski S, Krajewska M, Xie Z, Fuess S, Kitada S, Pawlowski K, Godzik A, Reed JC (2000). BAR: An apoptosis regulator at the intersection of caspases and Bcl-2 family proteins. Proceedings of the National Academy of Sciences of the United States of America.

[44] Metcalfe AD, Hunter HR, Bloor DJ, Lieberman BA, Picton HM, Leese HJ, Kimber SJ, Brison DR (2004). Expression of 11 members of the BCL-2 family of apoptosis regulatory molecules during human preimplantation embryo development and fragmentation. Molecular reproduction and development.

[45] Plotz M, Gillissen B, Quast SA, Berger A, Daniel PT, Eberle J (2013). The BH3-only protein Bim(L) overrides Bcl-2-mediated apoptosis resistance in melanoma cells. Cancer letters.

[46] Casanelles E, Gozzelino R, Marques-Fernandez F, Iglesias-Guimarais V, Garcia-Belinchon M, Sanchez-Osuna M, Sole C, Moubarak RS, Comella JX, Yuste VJ (2013). NF-kappaB activation fails to protect cells to TNFalpha-induced apoptosis in the absence of Bcl-xL, but not Mcl-1, Bcl-2 or Bcl-w. Biochimica et biophysica acta.

[47] Yin XM (2000). Bid, a critical mediator for apoptosis induced by the activation of Fas/TNF-R1 death receptors in hepatocytes. Journal of molecular medicine.

[48] Kodama T, Hikita H, Kawaguchi T, Shigekawa M, Shimizu S, Hayashi Y, Li W, Miyagi T, Hosui A, Tatsumi T, Kanto T, Hiramatsu N, Kiyomizu K, Tadokoro S, Tomiyama Y, Hayashi N (2012). Mcl-1 and Bcl-xL regulate Bak/Bax-dependent apoptosis of the megakaryocytic lineage at multistages. Cell death and differentiation.

[49] Huang J, Liu K, Song D, Ding M, Wang J, Jin Q, Ni J (2015). KLF4 promotes HMGB1-induced chemotherapy resistance in osteosarcoma cells. Cancer science.

[50] Bouralexis S, Findlay DM, Atkins GJ, Labrinidis A, Hay S, Evdokiou A (2003). Progressive resistance of BTK-143 osteosarcoma cells to Apo2L/TRAIL-induced apoptosis is mediated by acquisition of DcR2/TRAIL-R4 expression: resensitisation with chemotherapy. British journal of cancer.

[51] Baranski Z, Booij TH, Cleton-Jansen AM, Price LS, van de Water B, Bovee JV, Hogendoorn PC, Danen EH (2015). Aven-mediated checkpoint kinase control regulates proliferation and resistance to chemotherapy in conventional osteosarcoma. The Journal of pathology.

[52] Sevelda F, Mayr L, Kubista B, Lotsch D, van Schoonhoven S, Windhager R, Pirker C, Micksche M, Berger W (2015). EGFR is not a major driver for osteosarcoma cell growth *in vitro* but contributes to starvation and chemotherapy resistance. Journal of experimental & clinical cancer research : CR.

[53] Li S, Sun W, Wang H, Zuo D, Hua Y, Cai Z (2015). Research progress on the multidrug resistance mechanisms of osteosarcoma chemotherapy and reversal. Tumour biology : the journal of the International Society for Oncodevelopmental Biology and Medicine.

[54] Song B, Wang Y, Xi Y, Kudo K, Bruheim S, Botchkina GI, Gavin E, Wan Y, Formentini A, Kornmann M, Fodstad O, Ju J (2009). Mechanism of chemoresistance mediated by miR-140 in human osteosarcoma and colon cancer cells. Oncogene.

[55] Tian Y, Zhang YZ, Chen W (2014). MicroRNA-199a-3p and microRNA-34a regulate apoptosis in human osteosarcoma cells. Bioscience reports.

[56] Liu W, Xu G, Liu H, Li T (2015). MicroRNA-490-3p regulates cell proliferation and apoptosis by targeting HMGA2 in osteosarcoma. FEBS letters.

[57] Zhao H, Li M, Li L, Yang X, Lan G, Zhang Y (2013). MiR-133b is down-regulated in human osteosarcoma and inhibits osteosarcoma cells proliferation, migration and invasion, and promotes apoptosis. PloS one.

[58] Tian K, Di R, Wang L (2015). MicroRNA-23a enhances migration and invasion through PTEN in osteosarcoma. Cancer gene therapy.

[59] Maugg D, Rothenaigner I, Schorpp K, Potukuchi HK, Korsching E, Baumhoer D, Hadian K, Smida J, Nathrath M (2015). New small molecules targeting apoptosis and cell viability in osteosarcoma. PloS one.

[60] Daqian W, Chuandong W, Xinhua Q, Songtao A, Kerong D (2015). Chimaphilin inhibits proliferation and induces apoptosis in multidrug resistant osteosarcoma cell lines through insulin-like growth factor-I receptor (IGF-IR) signaling. Chemico-biological interactions.

[61] Wang XF, Wang J (2014). Icaritin suppresses the proliferation of human osteosarcoma cells *in vitro* by increasing apoptosis and decreasing MMP expression. Acta pharmacologica Sinica.

[62] Zhang Y, Wei RX, Zhu XB, Cai L, Jin W, Hu H (2012). Tanshinone IIA induces apoptosis and inhibits the proliferation, migration, and invasion of the osteosarcoma MG-63 cell line *in vitro*. Anti-cancer drugs.

[63] Yu X, Zhou X, Fu C, Wang Q, Nie T, Zou F, Guo R, Liu H, Zhang B, Dai M (2015). Celastrol induces apoptosis of human osteosarcoma cells via the mitochondrial apoptotic pathway. Oncology reports.

[64] Zhao X, Ma S, Liu N, Liu J, Wang W (2015). A polysaccharide from Trametes robiniophila Murrill induces apoptosis through intrinsic mitochondrial pathway in human osteosarcoma (U-2 OS) cells. Tumour biology : the journal of the International Society for Oncodevelopmental Biology and Medicine.

[65] Chen Y, Li M, Li Z, Gao P, Zhou X, Zhang J (2016). Bufalin induces apoptosis in the U2OS human osteosarcoma cell line via triggering the mitochondrial pathway. Molecular medicine reports.

[66] Ding L, He S, Sun X (2014). HSP70 desensitizes osteosarcoma cells to baicalein and protects cells from undergoing apoptosis. Apoptosis : an international journal on programmed cell death.

[67] Liu B, Shi ZL, Feng J, Tao HM (2008). Celecoxib, a cyclooxygenase-2 inhibitor, induces apoptosis in human osteosarcoma cell line MG-63 via down-regulation of PI3K/Akt. Cell biology international.

[68] Duan Z, Choy E, Harmon D, Yang C, Ryu K, Schwab J, Mankin H, Hornicek FJ (2009). Insulin-like growth factor-I receptor tyrosine kinase inhibitor cyclolignan picropodophyllin inhibits proliferation and induces apoptosis in multidrug resistant osteosarcoma cell lines. Molecular cancer therapeutics.

[69] Hanikoglu F, Cort A, Ozben H, Hanikoglu A, Ozben T (2015). Epoxomicin Sensitizes Resistant Osteosarcoma Cells to TRAIL Induced Apoptosis. Anti-cancer agents in medicinal chemistry.

[70] Li X, Huang T, Jiang G, Gong W, Qian H, Zou C (2013). Proteasome inhibitor MG132 enhances TRAIL-induced apoptosis and inhibits invasion of human osteosarcoma OS732 cells. Biochemical and biophysical research communications.

[71] Li M, Zhu Y, Zhang H, Li L, He P, Xia H, Zhang Y, Mao C (2014). Delivery of inhibitor of growth 4 (ING4) gene significantly inhibits proliferation and invasion and promotes apoptosis of human osteosarcoma cells. Scientific reports.

[72] Chipoy C, Brounais B, Trichet V, Battaglia S, Berreur M, Oliver L, Juin P, Redini F, Heymann D, Blanchard F (2007). Sensitization of osteosarcoma cells to apoptosis by oncostatin M depends on STAT5 and p53. Oncogene.

[73] Eliseev RA, Dong YF, Sampson E, Zuscik MJ, Schwarz EM, O'Keefe RJ, Rosier RN, Drissi MH (2008). Runx2-mediated activation of the Bax gene increases osteosarcoma cell sensitivity to apoptosis. Oncogene.

[74] Roos A, Satterfield L, Zhao S, Fuja D, Shuck R, Hicks MJ, Donehower LA, Yustein JT (2015). Loss of Runx2 sensitises osteosarcoma to chemotherapy-induced apoptosis. British journal of cancer.

[75] Guan H, Tan P, Xie L, Mi B, Fang Z, Li J, Yue J, Liao H, Li F (2015). FOXO1 inhibits osteosarcoma oncogenesis via Wnt/beta-catenin pathway suppression. Oncogenesis.

[76] Lu DF, Wang YS, Li C, Wei GJ, Chen R, Dong DM, Yao M (2015). Actinomycin D inhibits cell proliferations and promotes apoptosis in osteosarcoma cells. International journal of clinical and experimental medicine.

[77] Lv YF, Yan GN, Meng G, Zhang X, Guo QN (2015). Enhancer of zeste homolog 2 silencing inhibits tumor growth and lung metastasis in osteosarcoma. Scientific reports.

[78] Zhang Q, Padi SK, Tindall DJ, Guo B (2014). Polycomb protein EZH2 suppresses apoptosis by silencing the proapoptotic miR-31. Cell death & disease.

[79] Zhang Z, Shao Z, Xiong L, Che B, Deng C, Xu W (2009). Expression of Beclin1 in osteosarcoma and the effects of down-regulation of autophagy on the chemotherapeutic sensitivity. Journal of Huazhong University of Science and Technology Medical sciences = Hua zhong ke ji da xue xue bao Yi xue Ying De wen ban = Huazhong keji daxue xuebao Yixue Yingdewen ban.

[80] Liu J, Fan L, Wang H, Sun G (2016). Autophagy, a double-edged sword in anti-angiogenesis therapy. Medical oncology.

[81] Li S, Wang L, Hu Y, Sheng R (2015). Autophagy regulators as potential cancer therapeutic agents: a review. Current topics in medicinal chemistry.

[82] O'Farrill JS, Gordon N (2014). Autophagy in osteosarcoma. Advances in experimental medicine and biology.

[83] Sibirny AA (2011). Mechanisms of autophagy and pexophagy in yeasts. Biochemistry Biokhimiia.

[84] Rasmussen SB, Horan KA, Holm CK, Stranks AJ, Mettenleiter TC, Simon AK, Jensen SB, Rixon FJ, He B, Paludan SR (2011). Activation of autophagy by alpha-herpesviruses in myeloid cells is mediated by cytoplasmic viral DNA through a mechanism dependent on stimulator of IFN genes. Journal of immunology.

[85] Hurley JH, Schulman BA (2014). Atomistic autophagy: the structures of cellular self-digestion. Cell.

[86] Kumar D, Shankar S, Srivastava RK (2014). Rottlerin induces autophagy and apoptosis in prostate cancer stem cells via PI3K/Akt/mTOR signaling pathway. Cancer letters.

[87] de Iriarte Rodriguez R, Pulido S, Rodriguez-de la Rosa L, Magarinos M, Varela-Nieto I (2015). Age-regulated function of autophagy in the mouse inner ear. Hearing research.

[88] Kunchithapautham K, Rohrer B (2007). Apoptosis and autophagy in photoreceptors exposed to oxidative stress. Autophagy.

[89] Liu W, Shang G, Yang S, Huang J, Xue X, Lin Y, Zheng Y, Wang X, Wang L, Lin R, Tao J, Chen L (2015). Electroacupuncture protects against ischemic stroke by reducing autophagosome formation and inhibiting autophagy through the mTORC1-ULK1 complex-Beclin1 pathway. International journal of molecular medicine.

[90] Gammoh N, Florey O, Overholtzer M, Jiang X (2013). Interaction between FIP200 and ATG16L1 distinguishes ULK1 complex-dependent and -independent autophagy. Nature structural & molecular biology.

[91] Joassard OR, Amirouche A, Gallot YS, Desgeorges MM, Castells J, Durieux AC, Berthon P, Freyssenet DG (2013). Regulation of Akt-mTOR, ubiquitin-proteasome and autophagy-lysosome pathways in response to formoterol administration in rat skeletal muscle. The international journal of biochemistry & cell biology.

[92] Chen ZH, Cao JF, Zhou JS, Liu H, Che LQ, Mizumura K, Li W, Choi AM, Shen HH (2014). Interaction of caveolin-1 with ATG12-ATG5 system suppresses autophagy in lung epithelial cells. American journal of physiology Lung cellular and molecular physiology.

[93] Xia HG, Zhang L, Chen G, Zhang T, Liu J, Jin M, Ma X, Ma D, Yuan J (2010). Control of basal autophagy by calpain1 mediated cleavage of ATG5. Autophagy.

[94] Yin L, Liu J, Dong H, Xu E, Qiao Y, Wang L, Zhang L, Jia J, Li L, Geng X (2014). Autophagy-related gene16L2, a potential serum biomarker of multiple sclerosis evaluated by bead-based proteomic technology. Neuroscience letters.

[95] Suzuki M, Bartlett JD (2014). Sirtuin1 and autophagy protect cells from fluoride-induced cell stress. Biochimica et biophysica acta.

[96] Gruber HE, Hoelscher GL, Ingram JA, Bethea S, Hanley EN (2015). Autophagy in the Degenerating Human Intervertebral Disc: *In Vivo* Molecular and Morphological Evidence, and Induction of Autophagy in Cultured Annulus Cells Exposed to Proinflammatory Cytokines-Implications for Disc Degeneration. Spine.

[97] Geng J, Klionsky DJ (2008). The Atg8 and Atg12 ubiquitin-like conjugation systems in macroautophagy. ‘Protein modifications: beyond the usual suspects’ review series. EMBO reports.

[98] Miao XD, Cao L, Zhang Q, Hu XY, Zhang Y (2015). Effect of PI3K-mediated autophagy in human osteosarcoma MG63 cells on sensitivity to chemotherapy with cisplatin. Asian Pacific journal of tropical medicine.

[99] Liu Y, Zhao L, Ju Y, Li W, Zhang M, Jiao Y, Zhang J, Wang S, Wang Y, Zhao M, Zhang B, Zhao Y (2014). A novel androstenedione derivative induces ROS-mediated autophagy and attenuates drug resistance in osteosarcoma by inhibiting macrophage migration inhibitory factor (MIF). Cell death & disease.

[100] Meschini S, Condello M, Calcabrini A, Marra M, Formisano G, Lista P, De Milito A, Federici E, Arancia G (2008). The plant alkaloid voacamine induces apoptosis-independent autophagic cell death on both sensitive and multidrug resistant human osteosarcoma cells. Autophagy.

[101] Yang C, Shogren KL, Goyal R, Bravo D, Yaszemski MJ, Maran A (2013). RNA-dependent protein kinase is essential for 2-methoxyestradiol-induced autophagy in osteosarcoma cells. PloS one.

[102] Li X, Wang S, Chen Y, Liu G, Yang X (2014). miR-22 targets the 3′ UTR of HMGB1 and inhibits the HMGB1-associated autophagy in osteosarcoma cells during chemotherapy. Tumour biology : the journal of the International Society for Oncodevelopmental Biology and Medicine.

[103] Guo S, Bai R, Liu W, Zhao A, Zhao Z, Wang Y, Wang Y, Zhao W, Wang W (2014). miR-22 inhibits osteosarcoma cell proliferation and migration by targeting HMGB1 and inhibiting HMGB1-mediated autophagy. Tumour biology : the journal of the International Society for Oncodevelopmental Biology and Medicine.

[104] Huang J, Liu K, Yu Y, Xie M, Kang R, Vernon P, Cao L, Tang D, Ni J (2012). Targeting HMGB1-mediated autophagy as a novel therapeutic strategy for osteosarcoma. Autophagy.

[105] Yang C, Gao R, Wang J, Yuan W, Wang C, Zhou X (2014). High-mobility group nucleosome-binding domain 5 increases drug resistance in osteosarcoma through upregulating autophagy. Tumour biology : the journal of the International Society for Oncodevelopmental Biology and Medicine.

[106] Chang Z, Huo L, Li K, Wu Y, Hu Z (2014). Blocked autophagy by miR-101 enhances osteosarcoma cell chemosensitivity *in vitro*. TheScientificWorldJournal.

[107] Vicencio JM, Galluzzi L, Tajeddine N, Ortiz C, Criollo A, Tasdemir E, Morselli E, Ben Younes A, Maiuri MC, Lavandero S, Kroemer G (2008). Senescence, apoptosis or autophagy? When a damaged cell must decide its path--a mini-review. Gerontology.

[108] Huang WP, Klionsky DJ (2002). Autophagy in yeast: a review of the molecular machinery. Cell structure and function.

[109] He T, Wang HJ, Tan YZ (2008). [The roles of autophagy in cell survival and cell death]. Sheng li ke xue jin zhan [Progress in physiology].

[110] Das G, Shravage BV, Baehrecke EH (2012). Regulation and function of autophagy during cell survival and cell death. Cold Spring Harbor perspectives in biology.

[111] Zhang MS, Niu FW, Li K (2015). Proflavin suppresses the growth of human osteosarcoma MG63 cells through apoptosis and autophagy. Oncology letters.

[112] Li HY, Zhang J, Sun LL, Li BH, Gao HL, Xie T, Zhang N, Ye ZM (2015). Celastrol induces apoptosis and autophagy via the ROS/JNK signaling pathway in human osteosarcoma cells: an *in vitro* and *in vivo* study. Cell death & disease.

[113] Fan J, Yang X, Bi Z (2014). Acriflavine suppresses the growth of human osteosarcoma cells through apoptosis and autophagy. Tumour biology : the journal of the International Society for Oncodevelopmental Biology and Medicine.

[114] Wang Y, Ji Y, Hu Z, Jiang H, Zhu F, Yuan H, Lou H (2013). Riccardin D induces cell death by activation of apoptosis and autophagy in osteosarcoma cells. Toxicology *in vitro* : an international journal published in association with BIBRA.

[115] Zhang W, Li Q, Song C, Lao L (2015). Knockdown of autophagy-related protein 6, Beclin-1, decreases cell growth, invasion, and metastasis and has a positive effect on chemotherapy-induced cytotoxicity in osteosarcoma cells. Tumour biology : the journal of the International Society for Oncodevelopmental Biology and Medicine.

[116] Mori M, Hitora T, Nakamura O, Yamagami Y, Horie R, Nishimura H, Yamamoto T (2015). Hsp90 inhibitor induces autophagy and apoptosis in osteosarcoma cells. International journal of oncology.

[117] Akin D, Wang SK, Habibzadegah-Tari P, Law B, Ostrov D, Li M, Yin XM, Kim JS, Horenstein N, Dunn WA (2014). A novel ATG4B antagonist inhibits autophagy and has a negative impact on osteosarcoma tumors. Autophagy.

[118] Horie R, Nakamura O, Yamagami Y, Mori M, Nishimura H, Fukuoka N, Yamamoto T (2016). Apoptosis and antitumor effects induced by the combination of an mTOR inhibitor and an autophagy inhibitor in human osteosarcoma MG63 cells. International journal of oncology.

[119] Lee JW, Kim KS, An HK, Kim CH, Moon HI, Lee YC (2013). Dendropanoxide induces autophagy through ERK1/2 activation in MG-63 human osteosarcoma cells and autophagy inhibition enhances dendropanoxide-induced apoptosis. PloS one.

[120] Li X, Wu WK, Sun B, Cui M, Liu S, Gao J, Lou H (2011). Dihydroptychantol A, a macrocyclic bisbibenzyl derivative, induces autophagy and following apoptosis associated with p53 pathway in human osteosarcoma U2OS cells. Toxicology and applied pharmacology.

[121] Ji GR, Yu NC, Xue X, Li ZG (2015). PERK-mediated Autophagy in Osteosarcoma Cells Resists ER Stress-induced Cell Apoptosis. International journal of biological sciences.

[122] Trump BF, Berezesky IK, Chang SH, Phelps PC (1997). The pathways of cell death: oncosis, apoptosis, and necrosis. Toxicologic pathology.

[123] Pizarro A, Garcia-Tobaruela A, Herranz P, Pinilla J (1999). Thalidomide as an inhibitor of tumor necrosis factor-alpha production: a word of caution. International journal of dermatology.

[124] Liu M, Wu W, Li H, Li S, Huang LT, Yang YQ, Sun Q, Wang CX, Yu Z, Hang CH (2015). Necroptosis, a novel type of programmed cell death, contributes to early neural cells damage after spinal cord injury in adult mice. The journal of spinal cord medicine.

[125] Christofferson DE, Yuan J (2010). Necroptosis as an alternative form of programmed cell death. Current opinion in cell biology.

[126] Wu W, Liu P, Li J (2012). Necroptosis: an emerging form of programmed cell death. Critical reviews in oncology/hematology.

[127] Karl I, Jossberger-Werner M, Schmidt N, Horn S, Goebeler M, Leverkus M, Wajant H, Giner T (2014). TRAF2 inhibits TRAIL- and CD95L-induced apoptosis and necroptosis. Cell death & disease.

[128] Zhou W, Yuan J (2014). Necroptosis in health and diseases. Seminars in cell & developmental biology.

[129] Zhang YY, Liu H (2013). Connections between various trigger factors and the RIP1/RIP3 signaling pathway involved in necroptosis. Asian Pacific journal of cancer prevention : APJCP.

[130] Degterev A, Zhou W, Maki JL, Yuan J (2014). Assays for necroptosis and activity of RIP kinases. Methods in enzymology.

[131] Wu XN, Yang ZH, Wang XK, Zhang Y, Wan H, Song Y, Chen X, Shao J, Han J (2014). Distinct roles of RIP1-RIP3 hetero- and RIP3-RIP3 homo-interaction in mediating necroptosis. Cell death and differentiation.

[132] Kumari S, Redouane Y, Lopez-Mosqueda J, Shiraishi R, Romanowska M, Lutzmayer S, Kuiper J, Martinez C, Dikic I, Pasparakis M, Ikeda F (2014). Sharpin prevents skin inflammation by inhibiting TNFR1-induced keratinocyte apoptosis. eLife.

[133] Lamothe B, Lai Y, Xie M, Schneider MD, Darnay BG (2013). TAK1 is essential for osteoclast differentiation and is an important modulator of cell death by apoptosis and necroptosis. Molecular and cellular biology.

[134] Irrinki KM, Mallilankaraman K, Thapa RJ, Chandramoorthy HC, Smith FJ, Jog NR, Gandhirajan RK, Kelsen SG, Houser SR, May MJ, Balachandran S, Madesh M (2011). Requirement of FADD, NEMO, and BAX/BAK for aberrant mitochondrial function in tumor necrosis factor alpha-induced necrosis. Molecular and cellular biology.

[135] Moquin DM, McQuade T, Chan FK (2013). CYLD deubiquitinates RIP1 in the TNFalpha-induced necrosome to facilitate kinase activation and programmed necrosis. PloS one.

[136] Schenk B, Fulda S (2015). Reactive oxygen species regulate Smac mimetic/TNFalpha-induced necroptotic signaling and cell death. Oncogene.

[137] Zhu Y, Cui H, Gan H, Xia Y, Wang L, Wang Y, Sun Y (2015). Necroptosis mediated by receptor interaction protein kinase 1 and 3 aggravates chronic kidney injury of subtotal nephrectomised rats. Biochemical and biophysical research communications.

[138] Chromik J, Safferthal C, Serve H, Fulda S (2014). Smac mimetic primes apoptosis-resistant acute myeloid leukaemia cells for cytarabine-induced cell death by triggering necroptosis. Cancer letters.

[139] Duprez L, Bertrand MJ, Vanden Berghe T, Dondelinger Y, Festjens N, Vandenabeele P (2012). Intermediate domain of receptor-interacting protein kinase 1 (RIPK1) determines switch between necroptosis and RIPK1 kinase-dependent apoptosis. The Journal of biological chemistry.

[140] Zhou Z, Han V, Han J (2012). New components of the necroptotic pathway. Protein & cell.

[141] Fu Z, Deng B, Liao Y, Shan L, Yin F, Wang Z, Zeng H, Zuo D, Hua Y, Cai Z (2013). The anti-tumor effect of shikonin on osteosarcoma by inducing RIP1 and RIP3 dependent necroptosis. BMC cancer.

[142] Wada N, Kawano Y, Fujiwara S, Kikukawa Y, Okuno Y, Tasaki M, Ueda M, Ando Y, Yoshinaga K, Ri M, Iida S, Nakashima T, Shiotsu Y, Mitsuya H, Hata H (2015). Shikonin, dually functions as a proteasome inhibitor and a necroptosis inducer in multiple myeloma cells. International journal of oncology.

[143] Sun M, Zhou C, Zeng H, Puebla-Osorio N, Damiani E, Chen J, Wang H, Li G, Yin F, Shan L, Zuo D, Liao Y, Wang Z, Zheng L, Hua Y, Cai Z (2015). Hiporfin-mediated photodynamic therapy in preclinical treatment of osteosarcoma. Photochemistry and photobiology.

[144] Coupienne I, Fettweis G, Piette J (2011). RIP3 expression induces a death profile change in U2OS osteosarcoma cells after 5-ALA-PDT. Lasers in surgery and medicine.

